# Academic help seeking behavior as a mediator of the relationship between social skill and mathematics achievement among primary school students

**DOI:** 10.1038/s41598-025-99014-8

**Published:** 2025-06-04

**Authors:** Fasika Teshale Wole, Reda Darge Negasi, Amare Sahile Abebe

**Affiliations:** 1 Department of Psychology, College of Education & Behavioral Sciences, Bahir Dar University, Wollo University, Bahir Dar, Dessie, Ethiopia; 2 Department of Psychology, College of Education & Behavioral Sciences, Bahir Dar University, Bahir Dar, Ethiopia

**Keywords:** Academic help seeking, Mathematics achievement, Social skill, Psychology, Mathematics and computing

## Abstract

This study aimed to examine the mediating role of students’ academic help-seeking behavior in explaining the relationship between students’ social skills as motivational orientation and mathematics achievement among primary schools of Grade 8 students. Data were collected from 930 students among nine primary schools at the Dessie city administration in Ethiopia. The participant students were selected using systematic random sampling technique among selected schools. In line with the research objective, the research used correlational design in order to explain the relationship among variables. SPSS 24.0 and Smart PLS 4 were used for data analysis. The result of this study shows a significant direct positive effect of social skill on academic help-seeking behavior and mathematics achievement. Moreover, academic help-seeking behavior demonstrates a direct positive effect on mathematics achievement and plays a partial mediating role in the relationship between social skill and mathematics achievement. Thus, the findings indicated that the students’ academic help-seeking behaviors are part of the mechanism through which social skill affects students’ mathematics achievement. Accordingly, the research suggests the importance of comprehensive interventions to enhance students’ academic achievement.

## Introduction

In this dynamic and highly progressive world, those individuals who understand and can do mathematics will have significantly better opportunities and options for shaping their futures. Mathematical competence opens doors to productive futures. On the other hand, a lack of mathematical competence keeps those doorways closed^1,2^. Moreover, National Council of Teachers of Mathematics (NCTM) challenges the assumption that mathematics is only for a select few; rather, everyone needs to understand mathematics. All students should have the possibility and the assistance necessary to learn mathematics concepts with depth and understanding^2,3^.

Although there is global awareness of the importance of mathematical knowledge, research revealed that at various levels of education, students’ experience underachievement in mathematics^1,4^. Similarly, Abalde and Oco^5^ also explain that the study of mathematics at a different level of the educational hierarchy remains one of the most challenging, the prevalent students resulting in poor achievement in mathematics and related subjects. Accordingly, the performance of students’ in mathematics from primary school to higher education is still a topic of concern^1^.

In Ethiopia, the Ministry of Education (MoE) has been conducting national learning assessments (NLAs) every four year since 1999/2000. The result of the national learning assessment exclusively on the mathematics subject among Grade 8 students revealed that their average scores lay below the minimum expected threshold of 50% throughout the assessment years from 2000 to 2020. The national survey clearly indicates that there is a critical problem among Grade 8 students’ mathematics achievement at the national level^6^. As a result, the performance of students’ in mathematics from primary school to higher education is still a topic of concern.

Over the past three decades, researchers have succeeded in determining varieties of factors that significantly influenced students’ academic achievement in different subjects, especially in mathematics^1,7^. They revealed that among a variety of factors, enhancing the motivational conditions in their classroom for students’ is crucial for improving mathematics teaching and learning from its relationship with their behavior and achievement^8^. Motivational orientation plays a formative role in learning, acting as a potent fuel that directs, energizes, and regulates students’ behavior. Motivation enables the educators and researchers to get some clues to understand students’ behavior, efforts in doing various activities, and academic achievement^9^.

Regarding motivation at school, students’ social skills enable them to cooperate with their peers and actively engage in classroom learning activities, which could sustain attention and effort to learn that enhance academic achievement^10,11^. Researchers have demonstrated social skills as a separate, but important, influence on academic achievement. The way students behave in the classroom seems to directly contribute to how they learn and achieve. Studies have found that students who developed positive social skills were more successful in their roles as students, better positioned to engage in classroom settings, support each other, and better performed academically^12,13^.

More importantly, although extant research somewhat provides salient information regarding the role students’ social skills play in their academic achievement^13–15^; those previous investigations do not clearly show how social skills influence academic achievement. In particular, studies that look at the direct correlation between academic achievement and social skills lack the ability to identify the potential intervening factors in this relationship. Thus, the process in which social skills enhance academic achievement remains unclear^16^.

On the other hand, Caemmerer and Keith^16^ & DiPerna and Elliott^17^ suggested that social skills indirectly influence academic achievement by allowing students to more successfully work with peers, ask questions, listen, and attend to the classroom environment. Additionally, some studies have also evaluated the importance of social skills in order to explain students’ help seeking behavior^18,19^. However, still less research has established the nature of the relation between these constructs to enhance students’ academic achievement.

Consequently, the research has attempted to explain how students’ social skills influence their mathematics achievement. As a result, the research has proposed that students’ academic help seeking behavior may represent one of the key potential mechanisms that underlie the relationship between social skills and academic achievement. In support of this view, Patrick, Anderman^20^; Ryan and Shin^21^ indicated that social skills indirectly influence students’ academic achievement through enhancing behaviors that are more related to learning and achievement: academic help seeking behavior.

Over the last two decades, various researchers have shown that academic help seeking behavior is an adaptive learning strategy that is directly related to students’ learning and achievement^22,23^. On the other hand, they indicate that academic help seeking behavior is an inherently social process; social features of the learning context probably are more relevant in order to understand students’ academic help seeking behavior^23^. Students’ who have better social skills easily interact with others in the classroom to resolve their academic difficulties compared to those who struggle to cooperate with peers^10,23,24^.

Altogether, despite the existing literature on the relationship of these variables, several gaps remain in our understanding of how social skill, academic help seeking behavior and mathematics achievement interrelate. First, the inconsistent findings in the relationship between social skill and academic achievement might provide hints at mediating effects. Second, existing literature didn’t clearly show the mediating effect of academic help seeking behavior in the relationship between social skill and mathematics achievement. Therefore, the research was aimed at realizing the mediating role of academic help seeking behavior in explaining the relation between students’ social skills and their mathematics achievement among primary schools of Grade 8 students. Specifically, the following hypothesis was formulated and tested:-.

### H1

Student’s social skills have a significant positive impact on their mathematics achievement.

### H2

Student’s social skills have a significant positive impact on their academic help seeking behavior.

### H3

Student’s academic help seeking behavior has a significant positive impact on their mathematics achievement.

### H4

Student’s academic help seeking behavior partially mediated the relationship between social skills and their mathematics achievement.

## Literature review

### Theoretical framework

Social cognitive theory provides a theoretical and conceptual framework for the current study and helps explain how included constructs relate to one another. Social cognitive theory as developed by Bandura (1986) describes a triadic reciprocal relationship among someone’s personal characteristics, behaviors, and the environment^25^. The particular role and importance of any one of these three factors to cognitive development may change, given diverse sets of circumstances and conditions for every person, leading to many variations in how the triadic relationship manifests in different contexts^26^.

The reciprocal triadic relationship within social cognitive theory can be applied to understanding ways that individuals act and react to stimuli in their environments, thereby influencing behaviors and affecting learning^25^. Bandura’s social cognitive theory views students’ behavior and learning as a continuous interaction (reciprocal causation) between personal, behavioral, and environmental factors^25^. An important consideration within the social cognitive perspective is an acknowledgment that students’ academic achievement results from continuous, reciprocal interactions among behavior, the external environment, and cognitive factors^26^.

For several decades, researchers conducted studies on social cognitive learning theory emphasizing self-regulatory processes. Self-regulation focuses on the regulation of cognition and assesses the various cognitive and metacognitive strategies that students using^27^. In this view, academic help seeking considered a behavioral self-regulation strategy that students employ as they would cognitive and metacognitive strategies^18,28^. As strategy of self-regulation academic help seeking behavior enables students to properly use their environment for the benefit of their learning and achievement. Help-seeking exemplifies the critical role of social influences on learning and intellectual development^18,29^. However, help seeking behavior is unique as it involves social interaction among students; it is more likely affected by social feature of classroom. It needs students social competence (social skills), which might be perceived as incorporating the ideas of Bandura^28,29^.

Accordingly, students social behaviors are expected to play an essential role in order to understand their interaction, engage in learning activities, and decision of help seeking which, in turn, influences learning and achievement^30^. This makes a social cognitive framework a natural choice for understanding interaction between social skill, academic help seeking behavior, and academic achievement; importantly, how social skills influence academic achievement (Table 1).

### Empirical literature review

#### Social skills and academic achievement

Social skills such as cooperation with peers, initiating relationships, sharing and receiving compliments enable students to interact effectively with others and to avoid socially unacceptable responses^31^. Growing bodies of research continue to support the effect of students’ social skills on their academic achievement^32,33^. Regarding this, Malecki and Elliot^13^ found that social skills positively predicted current and future standardized academic achievement whereas problem behaviors negatively predicted current standardized academic achievement.

Considerable researchers have documented meaningful and predictive relationships between students’ social skill and academic achievement. They had been documented that students who have positive interactions and relationship with their peers are more academically engaged and have higher levels of academic achievement^34,35^. Particularly, during early adolescence period students’ identification with and conformity to peers increases dramatically; as a consequence, the quality of peer relationships at this age have a particularly strong impact on adjustment and subsequent achievement at school^13,36^.

#### Social skill and academic help seeking behavior

Social skills include the specific behaviors students have that allow them to effectively engage in a social task and promote positive interactions with others in their environment^12^. Within the classroom environment, that valued social skills are a key component and allow students to engage in reciprocal positive interactions with other students^37^. In support of this, Newman^38^ indicated that the social climate of the classroom, relationships with peers are important in order to understand students’ academic help-seeking behavior.

The existing limited researches shown that students who are better able to interact with others have more resources to benefit from the learning environment compared to students who struggle to cooperate with peers^24^. In line with this, Marchand and Skinner^39^ suggested that students would be more comfortable seeking help in classrooms characterized as caring, supportive, and friendly and where students felt that others know and related to them beyond their academic abilities. Students’ who are better able to interact with others have more resources to benefit from the learning environment compared to students’ who struggle to cooperate with peers^23,38^.

#### Academic help-seeking behavior and academic achievement

Help-seeking is also described as a strategy to overcome learning problems and promote mastery^18^. Using help-seeking, students will be able to recognize their learning problems and tackle those problems by asking questions from others^28^. Researchers have argued that help-seeking is adaptive for learning because the students are actively involved in the problem solving and the help merely serves as extra input for deep processing^23,30^.

An increase robust body of literature confirmed that academic help-seeking is an active strategy that serves as an aid to achieving academic success in the face of difficult or challenging tasks. They proved that students with higher adaptive-help seeking were more motivated and self-regulating, with higher course grades than those with higher avoidant of help seeking^22^. Moreover, Martin-Arbos, Castarlenas^40^ in a meta-analytic investigation attested that academic help-seeking self-regulated learning strategy which is directly related with students learning and achievement.

#### The mediating role of help seeking behavior

In the literature, the concept of academic help seeking behavior primarily guided by a perspective in which it is a behavioral self-regulated learning strategy, positively associated with students’ learning and achievement^18,40^. However, accumulated evidence indicates that students’ academic help seeking behavior is a complex process which is influenced by various motivational factors^23,39^. Since help seeking behavior is inherently social process; social features of the learning context probably are more relevant in order to explain students help seeking behavior^41^.

On the other hand, Caemmerer and Keith^16^ suggested that social skill indirectly influence achievement academic achievement by allowing students to more successfully work with peers, ask questions and attend to the classroom environment. Specifically, Marchand and Skinner^39^ & Newman^38^ indicated that students’ social skill influence their level of collaboration and help seeking behavior and, in turn, results in learning and achievement. Thus, it is plausible that students’ help seeking behavior might better explain the nature of relation between social skills with their mathematics achievement.

### Conceptual framework of the study

The most important point emerged from the literature, revealing that students’ social skills as motivational orientation have effects on academic achievement in school. However, the efficacy of the effect of social skill on academic achievement is questionable. Social skill might affect academic achievement, operating through academic help-seeking behavior, which is more related to students learning and achievement. Thus, the research hypothesized that students’ academic help-seeking behavior might mediate the relationship between students’ social skills and mathematics achievement in addition to the direct effect on their mathematics achievement.

**Fig. 1:**
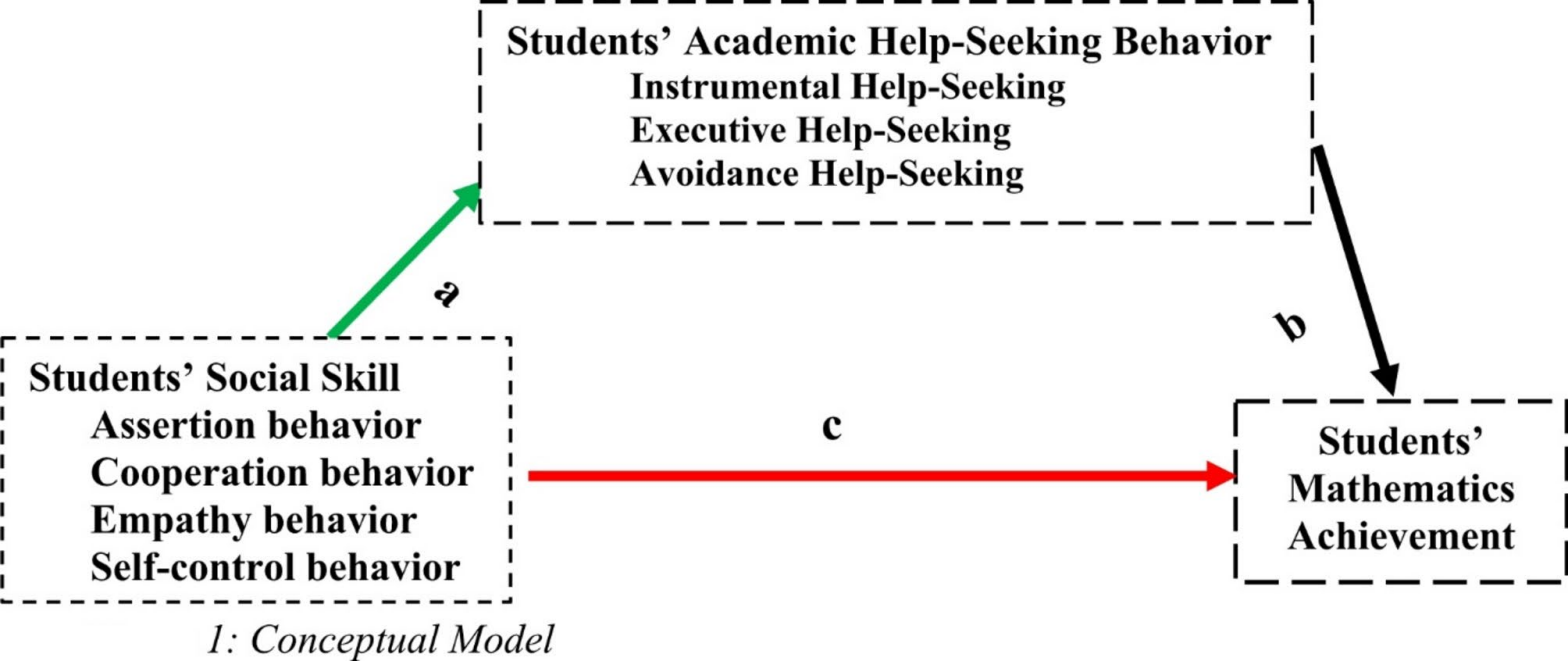
Conceptual model.

## Materials and methods

### Research design

In order to achieve the objective of the study, the research used correlational design. Correlational designs were provide an opportunity to predict scores and explain the relationship among variables. As Creswell^42^ explains, correlational designs are procedures in quantitative research in which investigators measure the degree of association (or relation) between two or more variables using the statistical procedure of correlational analysis. Thus, correlational design enables examining and understanding the nature of relationships among variables, which include the mediation role of academic help seeking behavior.

### Procedures for sampling and sampling techniques

The target populations of the study were students who registered for the **2023/24** academic year among randomly selected nine government primary schools of the Dessie city administration, Ethiopia. Consequently, the research was conducted exclusively among Grade 8 students from selected primary schools. The research had a total population of **1454** students among selected primary schools. Accordingly, the research adopted Stevens [1996 cited in 43] recommendations, which suggested having at least 15 cases per indicator. Thus, using the formula, among **1454** students, **930** were identified as a sample size for the research. By allocating a proportional number of students for each school, a systematic random sampling technique was employed to select participants from each school. It involves selecting subjects from a population list in a systematic random sampling method^43^. Among the total participants, 498 (53.5%) were male and 432 (46.5%) were female. To ensure ethical standards were met, respondent confidentiality and anonymity were maintained throughout the survey, participants were informed of the study’s purpose and their right to withdraw at any time, and informed consent was obtained from all participants.

### Measures

#### Social skill questionnaire

The study adapted the social skill questionnaires (SSQ) from Eslami, Amidi^44^, which are originally derived from the social skills rating systems child version (SSRS-C: Gresham & Elliot 1990). The scale consists of a total of 26 items, in which the cooperation and assertion subscales each contained 6 items, and the self-control and empathy subscales each contained 7 items. Items on these scales are rated on a three-point Likert-type scale with responses ranging from 1 = never to 3 = often. The internal consistency, construct, and predictive validity of this scale have been reported in previous research^31,44^. They indicated that the adapted instrument was found to be reliable. Accordingly, Eslami, Amidi^44^ reported that Cronbach’s alpha coefficients for the four subscales ranged from 0.72 to 0.83 (*p* < 0.001), in which self-control has 0.83, empathy has 0.78, assertion has 0.82, and cooperation has 0.72. Psychometric data for the present study, reported in Table 2 below, were good.

#### Academic help-seeking behaviors questionnaire

The academic help-seeking behaviors questionnaire (AHSBQ) was adapted from college algebra help seeking scales CAHSS^45^, which were originally designed to assess academic help-seeking behaviors of students enrolled in a computer science course^46^. The scale consists of three categories, namely instrumental, executive and avoidance of help seeking. It includes a total of 17 items in which instrumental and executive subscales each contained 5 items, and avoidance of help seeking contained 7 items. Items on these scales are rated on a five-point Likert-type scale with responses ranging from 1 = not at all true to 5 = very true). Higher scores indicate higher instrumental, executive, and avoidance of help seeking. The internal consistency, construct, and predictive validity of this scale have been reported in previous research^41,46^. They indicated that the adapted instrument was found to be reliable. Accordingly, Pajares, Cheong^46^ also found high Cronbach alphas coefficient of 0.89, 0.92, and 0.86 for the instrumental, executive, and avoidance of help Seeking scales, respectively. In addition, indices obtained from confirmatory factor analysis in this research (SRMR = 0.024, CFI = 0.97, TLI = 0.96, RMSEA = 0.055) indicated the appropriate fit of the model with the data. Psychometric data for the present study, reported in Table 2 below, were good.

#### Mathematics achievement

Students’ mathematics achievement was retrieved from official school documentation. Estimated results included all the assessments for the specified subject and were scaled at a maximum of 100, with higher scores reflecting better achievement. Then the researcher was considered students’ achievement score as a measure of their mathematics achievement.

### Data analysis techniques

Data analyses were performed using **IBM SPSS Statistics 24.0** (https://www.ibm.com/products/spss-statistics) and **SmartPLS 4** (https://www.smartpls.com/*)* software packages. SPSS 24.0 (Statistical Package for Social Sciences) is a windows based program which can be used to perform data entry and advanced statistical analysis^47^. Whereas, SmartPLS 4 is graphical user interface software using partial least squares (PLS) path modeling method. It enables researchers to investigate mediation and moderation effects, allowing them to delve deeper into understanding the underlying mechanisms in their models^48^. Accordingly, Partial least squares structural equations modeling (PLS-SEM) with bootstrap method was employed to evaluate the proposed model. PLS-SEM is a technique that enables to predict the behavior of the variables of the model proposed based on the theoretical review and understand a complex phenomenon which includes mediation relationships^48^. Therefore, first the confirmatory factor analysis (CFA) was performed to measure the model fit of each construct. Second, the reliability and validity of each scale were tested by providing the values of standardized factor load, composite reliability (CR), and Average Variance Extracted (AVE). Third, a correlation analysis was conducted to identify the direction of relevance and relationships among the variables. Finally, the proposed conceptual structural model was evaluated which includes direct and indirect relationships among variables.

#### Ethical aspects

The study has been evaluated and approved by the Institutional Research Review Committee of Bahir Dar University, Ethiopia, issuing an approval report: 00212. This study was conducted in accordance with the Declaration of Helsinki. The study respected the rights of the participants, as well as all the ethical principles of human research. Written informed consent was obtained from all of the participants and their parents before the data collection began. Permission to undertake the study was obtained from the officials of Dessie city administration.

## Results

### Preliminary data cleaning and assumptions testing

The final sample consisted of 924 participants, after removing 6 participants due to high percentages of missing data. Following deletion of these data, missing value analysis revealed no remaining missing data. The analysis of both univariate and multivariate outliers clearly indicated that there is no univariate outlier case as well as multivariate outlier as a result of the combination of scores. Analyses of normality make clear that nonnormality was not a problem. Moreover, the multicollinearity test result is also showing no significant multicollinearity issue.

### Measurement model

To assess measurements of model fit, reliability, and validity, confirmatory factor analysis (CFA) was conducted for the exogenous latent variable social skill, which has four dimensions, and the endogenous latent variable academic help-seeking behavior, which has three dimensions. The model fit was evaluated using five criteria. The model fit indices indicated that the data had acceptable fit to the measurement models for the exogenous latent variable: social skill (Chi-square/df = 2.59; SRMR = 0.025; CFI = 0.96; TLI = 0.95; RMSEA = 0.042) and the endogenous latent variable: academic help-seeking behavior (Chi-square/df = 3.77; SRMR = 0.024; CFI = 0.97; TLI = 0.96; RMSEA = 0.055)^49–51^. Table 1 summarizes the results of CFA model fit evaluation statistics for the measurement models.

**Table 1:** Model fit evaluation result for measurement.

	Model Fit Indices	Recommended cut off Criteria	Model 1	Model 2
1	Chisqr/df	< 5 (Schumacker & Lomax 2004)	2.59	3.77
2	SRMR	< 0.08 (Hu & Bentler, 1999)	0.025	0.024
3	CFI	> 0.90 (Hu & Bentler, 1999)	0.96	0.97
4	TLI	> 0.90 (Bentler, & Bonett,1980)	0.95	0.96
5	RMSEA	< 0.08 (Hu & Bentler, 1998)	0.042	0.055

Model 1 = Measurement model of social skill; Model 2 = Measurement model of Academic help seeking behavior.

Reliability refers to the consistency of scale tools. Cronbach’s alpha (CA) and composite reliability (CR) are used to measure reliability in this study. The CA value is between 0.87 and 0.93, whereas CR ranged from 0.90 to 0.96 (see Table 2). Both indicators of reliability have reliability statistics above the required threshold of 0.70, indicating that the data collected have good reliability^52^.

**Table 2:** Cronbach’s alpha, CR and AVE.

Scale	Dimension	Cronbach’s α	CR	AVE
AHSB	IHS	0.93	0.96	0.77
	EHS	0.86	0.90	0.64
	AHS	0.89	0.91	0.60
SS	CB	0.87	0.90	0.60
	AB	0.87	0.90	0.61
	SCB	0.89	0.92	0.61
	EB	0.88	0.91	0.59

IHS = Instrumental help seeking; EHS = Executive help seeking; AHS = Avoidance Help seeking; CB = Cooperation behavior; AB = Assertion behavior; SCB = Self-control behavior; EB = Empathy behavior.

The validity refers to the correctness of the scale tool, and the measurement indicators include convergent validity (CV) and discriminant validity (DV). Average variance extracted (AVE) is used to evaluate CV. The value of AVE for each construct ranges from 0.59 to 0.77, surpassing the minimum threshold of 0.50, suggesting that the measurement model has an acceptable CV^53^. With regard to the assessment of DV, Heterotrait-monotrait Ratio Statistics (HTMT**)** was employed. Accordingly, using the HTMT as a criterion involves comparing values to a predefined threshold, when the value of HTMT ratio of correlation is below 0.85 discriminant validity is established^54^. As shown in Table 3, the value of HTMT ratio of correlation is lower than the recommended threshold of 0.85; the discriminant validity is established.

**Table 3:** Discriminant validity test result: Heterotrait-monotrait ratio criterion (HTMT).

	AB	AHS	CB	EB	EHS	IHS	MA
AB							
AHS	0.132						
CB	0.444	0.034					
EB	0.460	0.163	0.575				
EHS	0.186	0.556	0.218	0.122			
IHS	0.451	0.579	0.554	0.572	0.226		
MA	0.505	0.512	0.519	0.593	0.242	0.671	
SCB	0.467	0.237	0.703	0.555	0.040	0.618	0.583

### Correlation analysis among the study variables

One of the objectives of the study was to examine the inter-correlations among dimensions of social skills (SS), academic help seeking behavior (AHSB), and mathematics achievement (MA). The findings found that all significant correlations were in theoretically expected directions. As indicated in Table 4 below, there was a significant positive correlation between dimensions of social skills (SS) with instrumental help seeking (IHS) and mathematics achievement (MA), indicating that better social skills were positively associated with both instrumental help seeking and subsequent mathematics achievement. However, there was a significant negative correlation between dimensions of social skills (SS) with executive help seeking (EHS) and avoidance of help seeking (AHS). Moreover, there was a significant negative correlation between executive help seeking (EHS) and avoidance of help seeking (AHS) with mathematics achievement (MA) (see Table 4).

**Table 4:** Inter-correlations among variables in the study.

	Variable	1	2	3	4	5	6	7
1	AB							
2	AHS	− 0.116**						
3	CB	0.385**	− 0.005					
4	EB	0.403**	− 0.144**	0.503**				
5	EHS	− 0.160**	− 0.47**	− 0.188**	− 0.107			
6	IHS	0.406**	− 0.526**	0.496**	0.518**	− 0.202**		
7	SCB	0.411**	− 0.211**	0.618**	0.493**	− 0.031	0.562**	
8	MA	0.471**	− 0.483**	0.482**	0.558**	− 0.225**	0.867**	0.551**

**p< 0.01 (2- tailed).

### Structural model analysis

The appropriateness of overall structural model was evaluated using indicators: SRMR, dULS and dG. The result indicated that the value of SRMR < 0.08 and dULS and dG are less than the 95% (**CI**_**95**_) quantile of their reference distribution^48^, demonstrating an acceptable fit of the model (see Table 5). This implies that results obtained from the SEM analysis are more precise and reliable to determine mathematics achievement.

**Table 5:** Overall models fit evaluation result.

Model Fit Indices	Value	CI_95_	Conclusions
Saturated Model			
SRMR	0.072	0.074–0.076	Supported
d_ULS_	0.852	0.910–0.924	Supported
d_G_	0.302	0.305–0.310	Supported
Estimated Model			
SRMR	0.075	0.076–0.079	Supported
d_ULS_	1.011	1.024–1.130	Supported
d_G_	0.254	0.298–0.306	Supported

SRMR = standardized residual mean square root, d_ULS_ = distance of unweighted least squares and d_G_ = geodesic distance.

Partial least squares structural equation modeling (PLS-SEM) with bootstrapping estimation procedures was used to evaluate the proposed model. The standardized path coefficients were examined using Keith (2019) evaluation criteria. As an approximate guide, Keith recommends that β > 0.05 are considered as small, β > 0.10 moderate, and β > 0.25 large^55^.

**Fig. 2:**
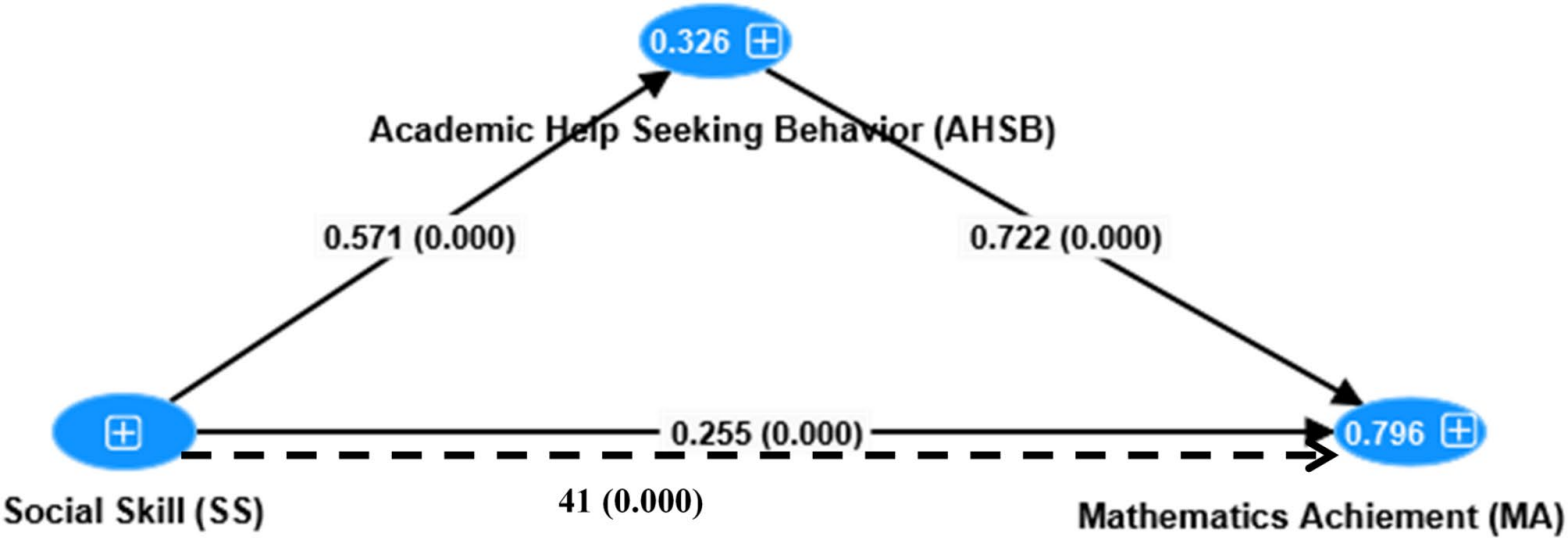
Analytic diagram for proposed mediation model (broken-line represent indirect relations).

### Direct effect analysis

As shown in the Table 6 in SEM path model mathematics achievement was directly predicted by social skill (β= 0.26, *p* < 0.001, 95%CIs [0.21, 0.30]) which excluding 0 and academic help seeking behavior (β= 0.72, *p* < 0.001, 95%CIs [0.68, 0.76]) which excluding 0 which was above variance accounted by social skill. Moreover, social skill was directly predicted academic help seeking behavior (β= 0.57, *p* < 0.001, 95%CIs [0.52, 0.61]) which excluding 0 (see Table 6).

**Table 6:** Test results of the proposed direct effect.

Hypothesis	Relationship	Path Coefficient	Confidence Interval		Remarks
			LB	UB	
H1:	**SS → MA**	0.26***	0.21	0.30	Supported
H2:	**SS → AHSB**	0.57***	0.52	0.61	Supported
H3:	**AHSB → MA**	0.72***	0.68	0.76	Supported

*** *P* < 0.001.

### Mediating effect of academic help seeking behavior

The mediating model (Fig. 1) included a path that indicated the indirect effects of social skill on students’ mathematics achievement through academic help seeking behavior. SmartPLS 4.0 was used to examine the meditational model. A sample size of b = 5,000 was selected for random samplings to calculate indirect effects using a bias-corrected bootstrapping procedure. The mediation effect is considered significant if the 95% bias-corrected confidence interval (BCCI) does not include 0. The findings indicate that the indirect effect path (SS **→** AHSB → MA) is significant (β = 0.41, 95% CI = [0.38, 0.45]). Moreover, with the inclusion of the mediator the effect of **SS** on **MA** was still significant (see Fig. 2); AHSB was partially mediating the relation between **SS** and **MA**. Mediation summery is presented in Table 7.

**Table 7:** Mediating effect analysis summary.

Hypothesis	Relationship	Direct Effect	Indirect Effect	Confidence Interval		Test Results
				LB	UB	
H4:	**SS – AHSB – MA**	0.26***	0.41***	0.38	0.45	Supported

*** *P* < 0.001.

## Discussion

This study examined the correlation among variables, direct and indirect effects of students’ social skill on their mathematics achievement among primary schools of Grade 8 students (see Fig. 2). The major findings were as follows.

### Direct relationships

The results from a structural equation model concerning the direct role of social skill on academic help seeking behavior and mathematics achievement and academic help seeking behavior on mathematics achievement established the following facts in each path.

The results of the study confirmed that the students’ social skills significantly and positively influenced the mathematics achievement of primary school students, which is consistent with the findings of previous studies^12,14,35,56^. These studies demonstrate that social skills have an important behavioral factor that influences the students’ academic achievement. They demonstrated that social skills are a separate but important influence on academic achievement. In support of social skills on math achievement Malecki and Elliot^13^ found a significant relationship between teaching ratings, social skills rating systems, and math achievement.

The result of the study supports the hypothesis that students’ social skills are an important motivational factor creating an encouraging learning environment that influences their academic achievement. Students who have positive interactions and relationships with their peers are more academically engaged and have higher levels of academic achievement^20,34,35^. These studies specified that the quality of students’ social relationships has a motivational significance, creating contexts that make students feel like they are a valued part of the classroom, hence making students more likely to adopt positive learning behaviors that lead to academic achievement.

Further, regarding the role of social skill on academic help seeking behavior, the results affirm that social skill significantly and positively predicted changes in help-seeking behavior among primary school Grade 8 students. Consistent with this finding, Elliott, Malecki^37^ explain that within the classroom environment, social skills are a key component and allow students to engage in reciprocal positive interactions with their peers. If students need help with their schoolwork but do not seek it, then their lack of social interaction in the classroom. When mutual respect and caring are prevalent in the classroom, students may be less likely to take unfair advantage of their peers^21,39^.

The results affirm the motivational and affective significance of social skill on students’ help seeking behavior. Social interactions in the classroom exert a powerful influence on student motivation, engagement, and learning. It determines their level of cooperation and help seeking behavior to resolve academic difficulties when they need^30^. A caring, respectful, and supportive peer climate is also likely to promote adaptive help seeking. Students’ who are better able to interact with others are more motivated and have more resources to benefit from the learning environment compared to students’ who struggle to cooperate with peers^21,24,57^.

Finally, the predictive model consistent with previous findings attested that academic help seeking behavior significantly and positively predicted mathematics achievement of primary school Grade 8 students^21,22^, indicating that students’ help-seeking behavior significantly predicted changes in their academic achievement. Researches specified that engaging in adaptive help seeking is conducive to learning, which enhances academic achievement, as it involves requests for help that would further learning and promote independent problem solving^40^.

The result supports the important role of help-seeking to avoid potential failure and increase the likelihood of achievement^23^. In the academic context, students who seek help with coursework are better able to maintain involvement in difficult tasks, avoid the possibility of academic failure, and increase their chances of mastering the material^28^. Using help-seeking, students will be able to recognize their learning problems and tackle those problems by asking questions of others. The research makes clear that help seeking is an active strategy that serves as an aid to achieve academic success in the face of academic difficulty^21,23,28^.

### Mediating relationship

Although social skill has been studied thoroughly, it often does not address how social skill functions in order to enhance students’ academic achievement. Thus, academic help seeking behavior was tested to mediate the relationship between social skill and mathematics achievement. As one of the few studies Caemmerer and Keith^16^, Patrick, Anderman^20^ that stated that social skill indirectly influences academic achievement by allowing students to more successfully work with peers, ask questions, listen, and attend to the classroom environment. The results confirmed that a student’s social skill indirectly influences mathematics achievement through its effect on their help seeking behavior.

Accordingly, the mediating role of help seeking behavior found in this study supports the theoretical explanations of bandura’s social cognitive perspective, which adopts a cognitive and motivational perspective towards identifying the determinants of behavior^25^; academic achievement results from continuous, reciprocal interactions among behavior (e.g., social skills), the external environment, and cognitive factors^58^. Thus, the finding highlights that students’ help seeking behavior is one of the mechanisms through which social skills influence their mathematics achievement.

The results indicated that students having better social skills tend to show supportive relationships with their peers, which lays a solid foundation for their preference of seeking help behavior^20,59^, which, in turn, are related to their academic achievement. This finding also shows those students who describe having positive, intimate and supportive relationships with their peers in the classroom possess adaptive help seeking behavior. Such students feel less anxious and feel confident about seeking help and actively support each other to resolve their learning difficulties in learning math, which in turn influence their mathematics achievement.

By evaluating the mediational effect of students’ academic help seeking behavior the study extent previous findings Gresham^12^, DiPerna^14^, Miles and Stipek^35^ which examined the direct link between students’ social skill and academic achievement; the result verified that the association between social skill and achievement was mediated by students’ help seeking behavior. This implies that social skills (students’ interaction with their peers) in the classroom enhanced the role of academic help seeking behavior on their mathematics achievement in the context of primary school education.

## Conclusion

This study empirically examined the relationship between social skill, academic help seeking behavior, and mathematics achievement among primary school Grade 8 students. Based on the previous findings in the literature about mathematics achievement, the present research has proposed and tested the hypothesis by collecting data from nine randomly selected primary schools in Dessie, Ethiopia.

The results showed that students’ social skills were positively and significantly predicted their academic help seeking behavior, and mathematics achievement. Accordingly, the findings suggested the motivational and affective significance of students’ social skills on their help seeking behavior and mathematics achievement. Moreover, the result has confirmed that students’ academic help seeking behavior had a significant and positive effect on their mathematics achievement and plays a mediating role in the relationship between their social skill and mathematics achievement. Furthermore, the mediational study found that academic help seeking behavior looks more like academic related behavior, which influences students’ learning and mathematics achievement.

In general, the study suggests that social skills are important, not just because of the way that they are directly related to academic achievement, but also because of the ways in which they promote or inhibit students’ academic help seeking behavior, in turn enhancing their mathematics achievement. Thus, researchers and educators need to consider carefully how best to boost students’ social skills in the classroom in order to enhance their academic help seeking behavior, and ultimately their mathematics achievement.

### Contributions

This study makes three important contributions to the literature on the relationship between social skill, academic help seeking behavior and mathematics achievement among primary school students. First, the study revealed students social skill has important contribution to their mathematics achievement and academic help seeking behavior. Second the study found the significance role of academic help seeking behavior on students’ mathematics achievement which enriches existing scant studies in these areas. Lastly, the study considers how social skills influence students’ mathematics achievement. The study shows that academic help seeking behavior is one of the mechanisms through which social skills influence mathematics achievement. Previous studies have rarely focused on studying the mechanisms underlying the effects of social skill on academic achievement^16,17^.

Altogether, results from such analysis inform both theoretical and practical aspects. Theoretically, it provides information on the relative importance of the mediator, which further improves theorizing the mechanisms of influence. Practically, teaching programs should promote learning environments that encourage social skills and help seeking among peers which ultimately boosted academic achievement. Moreover, the results suggest the importance of comprehensive interventions for an optimal outcome. In conclusion, assessing the mechanisms through which social skills influence primary school students’ mathematics achievement is important because multitudes of variables may intervene in the process, which, if not detected, would lead to erroneous conclusions regarding direct effects.

## Limitations and future research directions

This study had certain limitations, and it should be noted for future research. First, we only investigated the mathematics achievement of primary school Grade 8 students from the Dessie city of Ethiopia. Future studies could attempt to replicate and verify our findings using a more extensive survey. Second, we explored the mediating role of academic help seeking behavior in the effects of social skill on achievement in mathematics using a cross-sectional study design. Longitudinal studies or interviews could be included in future studies. Finally, given the challenges learning mathematics and the presence of strong peer interaction at this stage of development, we hope future work will continue to advance understanding the role of social skills on students’ motivation and achievement, and the important contribution of help seeking behavior among peers in order to solve learning difficulties & boosted their academic achievement. Importantly, how social skills influence academic achievement among primary schools.

## Data Availability

The raw data supporting the conclusion of this article will be made available on request from the corresponding authors.
